# Distribution of Birth Weight for Gestational Age in Japanese Infants Delivered by Cesarean Section

**DOI:** 10.2188/jea.JE20100123

**Published:** 2011-05-05

**Authors:** Ritei Uehara, Fumihiro Miura, Kazuo Itabashi, Masanori Fujimura, Yosikazu Nakamura

**Affiliations:** 1 Department of Public Health, Jichi Medical University, Tochigi, Japan; 2 Department of Pediatrics, Showa University School of Medicine, Tokyo, Japan; 3 Osaka Medical Center and Research Institute for Maternal and Child Health, Osaka, Japan

**Keywords:** birth weight, distribution, gestational age, cesarean section, preterm

## Abstract

**Background:**

Neonatal anthropometric charts of the distribution of measurements, mainly birth weight, taken at different gestational ages are widely used by obstetricians and pediatricians. However, the relationship between delivery mode and neonatal anthropometric data has not been investigated in Japan or other countries.

**Methods:**

The subjects were selected from the registration database of the Japan Society of Obstetrics and Gynecology (2003–2005). Tenth centile, median, and 90th centile of birth weight by sex, birth order, and delivery mode were observed by gestational age from 22 to 42 weeks among eligible singleton births.

**Results:**

After excluding 248 outliers and 5243 births that did not satisfy the inclusion criteria, 144 980 births were included in the analysis. The distribution of 10th centile curves was skewed toward lower birth weights during the preterm period among both first live births and second and later live births delivered by cesarean section. More than 40% of both male and female live births were delivered by cesarean section at 37 weeks or earlier.

**Conclusions:**

The large proportion of cesarean sections influenced the skewness of the birth weight distribution in the preterm period.

## INTRODUCTION

Neonatal anthropometric charts are based on the distribution of measurements, mainly birth weight, of neonates at different gestational ages.^1^ The Japanese neonatal anthropometric charts, which were revised in 1995,^2^ are widely distributed to Japanese obstetricians and pediatricians for managing pregnancy and newborns.

Because more than 10 years had passed since publishing the revised charts, the research committee of the Ministry of Health, Welfare, and Labour for Multicenter Benchmark Research on Neonatal Outcomes in Japan attempted to develop new anthropometric charts. Due to the small sample size, the 1995 charts only contained data classified by sex and birth order. Using the registration database of the Japan Society of Obstetrics and Gynecology (JSOG), which includes a large number of pregnant women and their babies, we attempted to construct charts by mode of delivery, ie, vaginal delivery and cesarean section, as well as sex and birth order. This delivery mode-specific chart is unique to Japan, as no such chart exists in other countries.^3^^–^^7^ In this study, we describe the different birth-weight distributions by gestational age and mode of delivery and discuss the factors that influenced this distribution.

## METHODS

JSOG manages a registration system for pregnant women and their infants. To construct new neonatal anthropometric charts, we collected data from 2003 to 2005 on gestational age, birth weight, sex, birth order, and information on complications of singleton births from this database. Because JSOG approved the use of their database for the purpose of creating new neonatal anthropometric charts, this study was not subject to institutional review. Stillborn infants and those with severe asphyxia (Apgar score of 0 at 1 and 5 minutes after delivery), hydrops, or malformations were excluded from the analysis. Infants with missing information on sex or gestational age were also excluded.

Regarding mode of delivery, 6 modes were reported in the registration database: natural vaginal delivery, vacuum-assisted vaginal delivery, forceps-assisted vaginal delivery, elective cesarean section, emergency cesarean section, and others. Natural vaginal delivery, vacuum-assisted vaginal delivery, and forceps-assisted vaginal delivery were defined as vaginal delivery, and elective and emergency cesarean sections were defined as cesarean delivery in this study. Because more than 80% of births delivered by elective cesarean section were delivered from 37 to 41 gestational weeks and approximately 60% of those delivered by emergency cesarean section were delivered at 36 week or earlier, we combined these modes of delivery in the analysis. Pregnant women for whom mode of delivery was classified as “others” were excluded from this analysis.

First, 10th centile, median, and 90th centile of birth weight by sex and birth order (first live births or second and later live births) were observed by gestational age from 22 to 42 weeks among all eligible births. Then, a similar observation was made by delivery mode. The values obtained were then plotted and fitted to cubic curves using the least squares method.

## RESULTS

During the study period, 147 medical facilities participated in the JSOG registration system, and 150 471 singleton births were reported to the registration database. A total of 5243 births were excluded from the analysis; thus, the study population comprised 145 228 births. Then, an additional 248 clinical outliers were excluded from this population. Consequently, 144 980 singleton births (74 740 boys and 70 240 girls) were included in the analysis (Table 1). Among the 74 740 boys, 39 707 were first live births and 35 033 were second or later live births. Among the 70 240 girls, 36 827 and 33 413 were first live births and second or later live births, respectively.

**Table 1.** Number of singleton births by gestational week and birth order, 2003–2005

Gestational week	Male			Female		
	
	First live births	Second and later live births	Total	First live births	Second and later live births	Total
22	26	30	56	21	30	51
23	76	63	139	48	52	100
24	92	107	199	73	84	157
25	103	129	232	96	125	221
26	140	122	262	97	152	249
27	156	185	341	135	129	264
28	203	202	405	151	175	326
29	197	209	406	161	170	331
30	252	234	486	228	222	450
31	273	304	577	236	235	471
32	393	417	810	300	325	625
33	502	486	988	381	382	763
34	741	653	1394	517	533	1050
35	944	876	1820	724	680	1404
36	1537	1428	2965	1240	1262	2502
37	3720	5083	8803	3306	4561	7867
38	6691	8126	14 817	5751	7424	13 175
39	9698	8301	17 999	8795	7846	16 641
40	9271	6129	15 400	9446	6756	16 202
41	4463	1894	6357	4812	2186	6998
42	229	55	284	309	84	393

Total	39 707	35 033	74 740	36 827	33 413	70 240

**Figure 1.**
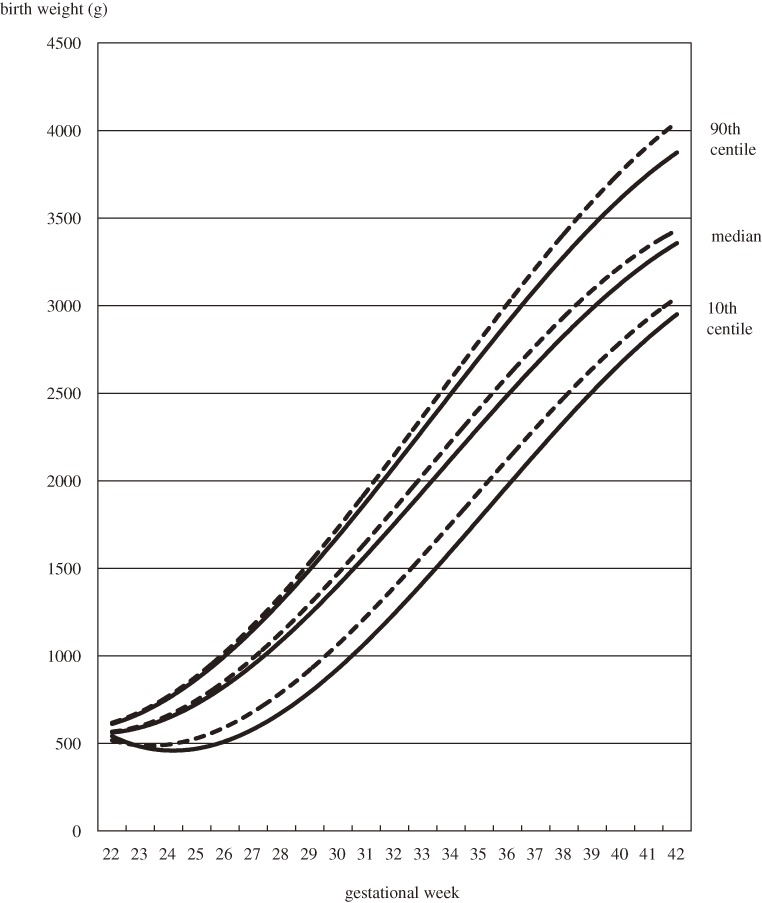
Distribution of birth weights of singleton males by gestational age and birth order, 2003–2005. Cubic curves were drawn using the least squares method. Solid lines show first live births; dotted lines show second and later live births.

**Figure 2.**
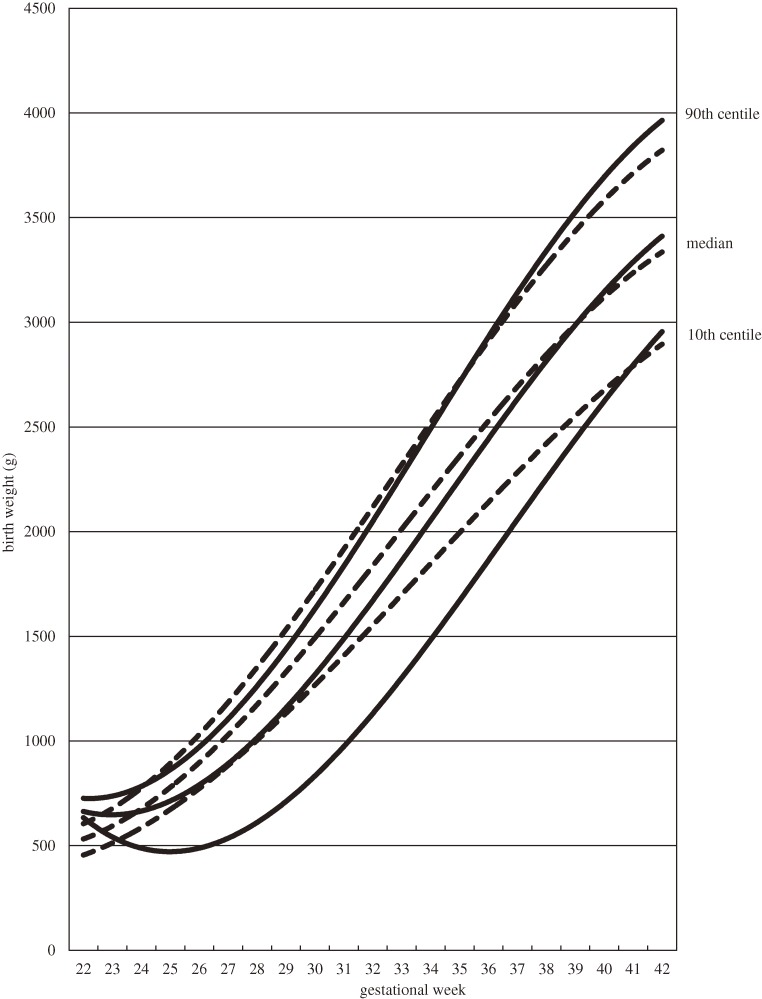
Distribution of birth weights of first live male births by gestational age and delivery mode, 2003–2005. Cubic curves were drawn using the least squares method. Solid lines show births by cesarean section; dotted lines show births by vaginal delivery.

**Figure 3.**
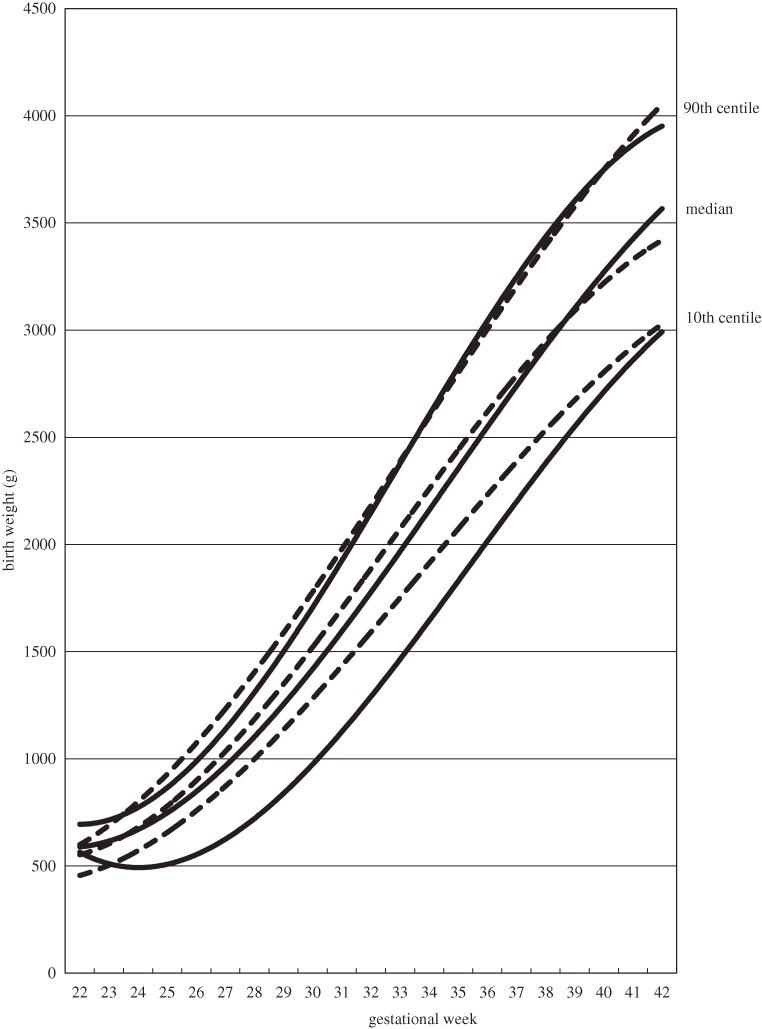
Distribution of birth weights of second and later live male births by gestational age and delivery mode, 2003–2005. Cubic curves were drawn using the least squares method. Solid lines show births by cesarean section; dotted lines show births by vaginal delivery.

Figure 1 shows the birth weight distribution of singleton male infants by gestational age and birth order. The 10th centile curves of first live births and second and later live births were skewed to lower birth weights in the preterm period. When the birth weight distributions are classified by delivery mode, the 10th centile curves were skewed to lower birth weights among both first live births and second and later live births delivered by cesarean section (Table 2, Figures 2 and 3). Coefficients of determination of all fitted curves were higher than 0.98, and the skewness was similar in 10th centile curves of birth weight of female infants who were delivered by cesarean section (data not shown).

**Table 2.** Tenth centile, median, and 90th centile of birth weights of singleton males by gestational week and birth order, 2003–2005

Gestational week	Vaginal delivery (g)			Cesarean delivery^a^ (g)		
	
	10th centile	Median	90th centile	10th centile	Median	90th centile
First live births						
22	443	507	558	—	—	—
23	520	602	674	505	594	670
24	589	680	769	470	637	798
25	619	784	976	422	718	862
26	806	900	1026	544	864	1014
27	928	1060	1182	650	980	1158
28	1038	1156	1379	678	1056	1342
29	1093	1371	1542	689	1147	1430
30	1270	1510	1688	830	1325	1618
31	1408	1638	1864	941	1402	1794
32	1546	1774	2076	1118	1638	2000
33	1731	2000	2356	1260	1834	2236
34	1834	2190	2513	1406	2010	2444
35	1944	2338	2694	1558	2176	2664
36	2050	2508	2912	1760	2406	2930
37	2272	2714	3142	2200	2719	3170
38	2460	2876	3308	2374	2880	3360
39	2632	3025	3446	2500	3026	3618
40	2728	3142	3580	2663	3221	3770
41	2815	3234	3686	2796	3297	3848
42	2816	3297	3818	2858	3311	3863
Second and later live births						
22	458	513	590	546	570	593
23	460	594	678	450	596	774
24	594	682	800	481	658	770
25	684	805	899	572	798	915
26	724	960	1120	648	856	1018
27	870	1050	1270	584	996	1179
28	1092	1226	1492	732	1134	1348
29	1142	1334	1510	936	1296	1536
30	1223	1513	1779	990	1384	1682
31	1487	1680	1916	1160	1580	1880
32	1569	1864	2200	1180	1727	2082
33	1732	2040	2388	1388	1865	2236
34	1910	2204	2524	1530	2162	2582
35	1985	2378	2750	1660	2318	2830
36	2170	2610	3054	2001	2580	3102
37	2405	2822	3270	2380	2820	3275
38	2595	3006	3442	2566	2982	3466
39	2734	3145	3584	2602	3120	3648
40	2850	3270	3742	2692	3265	3773
41	2940	3372	3830	2824	3366	3976
42	2950	3308	4080	2926	3567	3800

^a^There were no eligible first live male births born by cesarean section in gestational week 22.

Values were plotted and fitted to cubic curves by using the least squares method in Figures 2 and 3.

**Figure 4.**
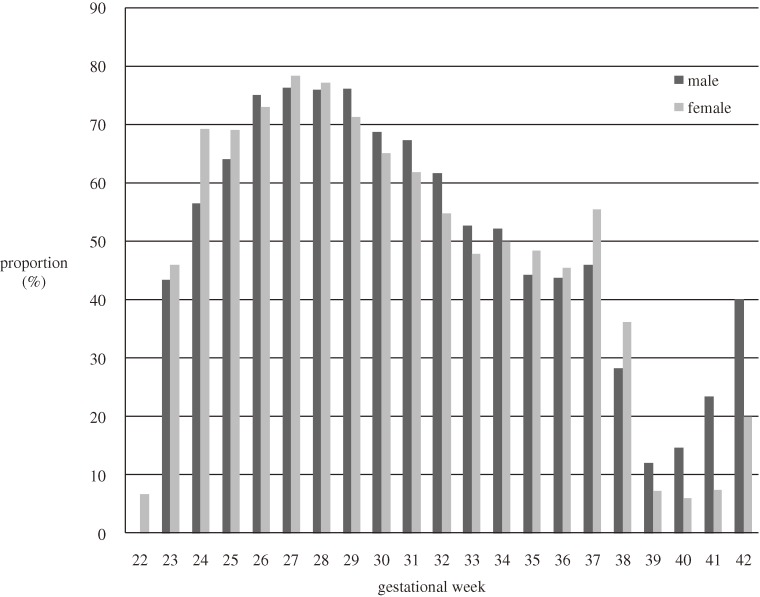
Proportion of first live cesarean section births by gestational age, 2003–2005.

The proportion of first live births delivered by cesarean section by gestational age is shown in Figure 4. More than 40% of male and female births were delivered by cesarean section at 37 weeks or earlier. From 26 to 29 weeks, more than 70% of births were delivered by cesarean section.

## DISCUSSION

The 10th centile birth weight curves of Japanese singleton infants by gestational age were skewed toward low values during the preterm period. Cesarean section influenced this distribution because the proportion of births delivered by cesarean section was large during the preterm period, especially from 26 to 29 weeks. As curves for 24 to 26 gestational weeks appeared to be markedly skewed toward low values, there was a difference in gestational period between the area of the curves with the most skewness and that representing the largest proportion of cesarean sections. We were unable to determine the reason for this, as no country has included delivery mode in neonatal anthropometric charts.^2^^–^^7^ Due to this uncertainty, the research committee for creating new neonatal anthropometric charts in Japan decided to eliminate cesarean deliveries from the charts. The new Japanese neonatal anthropometric chart will thus include only the birth weight of singleton infants born by vaginal delivery as standard curves, which are created after excluding factors related to fetal growth. We used the least squares methods to calculate the distribution of birth weights in this study because it was also employed in the revised charts in 1995.^2^ The LMS (λ, μ, σ) method, however, will be used to create the new Japanese charts.^8^

More than 40% of preterm infants were delivered by cesarean section in Japan. The proportion of cesarean sections was reported to be increasing among preterm infants in the United States.^9^^–^^11^ The reasons for cesarean section are not available in the JSOG registration database; however, one known reason is fetal growth restriction (FGR), which is a decrease in the fetal growth rate that inhibits an infant from obtaining its complete genetic growth potential. FGR is caused by placental dysfunction or maternal complications such as pre-eclampsia.^12^^,^^13^ It is associated with increased perinatal mortality and morbidity, as well as with increased risk of long-term complications such as impaired neurodevelopment, adult type 2 diabetes, and hypertension.^13^ Ultrasonography techniques, including the non-stress test, biophysical profile scoring, and pulse Doppler methods, enable obstetricians to carefully evaluate fetal growth.^14^ Due to these methods of fetal management, especially observation of growth in fetal head circumference, obstetricians are more likely to deliver fetuses with FGR during preterm in the event of non-reassuring fetal status. Indeed, approximately 80% of fetuses with FGR were delivered by cesarean section in European countries.^15^ Cesarean section is also likely to be selected in cases of preterm premature rupture of membranes.^16^

Because the JSOG database mainly includes tertiary hospitals, low birth weight infants were overrepresented in our study population as compared with the general population. It has been reported that whereas 8.5% of male births and 10.8% of female births were less than 2500 grams in the general population, approximately 25% of births were less than 2500 grams in some tertiary hospitals.^17^^–^^19^ In addition, pregnant women with complications might be more likely to be admitted to, and undergo cesarean section in, tertiary hospitals. Due to this selection bias, 10th centile birth weights of cesarean section births may be less than those of the general population. The reliability of gestational age is the most important issue in creating neonatal anthropometric charts. We were unable to confirm whether gestational age was assessed by ultrasonography during first trimester among pregnant women registered in the JSOG system. Many Japanese clinics and hospitals that treat pregnant women have ultrasonography equipment. However, because estimation of gestational age by ultrasonography was not mentioned in Japanese guidelines for obstetrical practice, some facilities may have calculated gestational age by asking pregnant women about their last menstrual period.^20^

In conclusion, the 10th centile birth weight curves of Japanese singleton infants delivered by cesarean section by gestational age were skewed toward low values during the preterm period. This might reflect the fact that fetuses with FGR were more likely to be delivered by cesarean section to prevent worsening fetal growth. Thus, the birth weights of singleton infants born by vaginal delivery were used as standard curves to develop new Japanese neonatal anthropometric charts.
